# Viral dynamics and antibody responses in people with asymptomatic SARS-CoV-2 infection

**DOI:** 10.1038/s41392-021-00596-2

**Published:** 2021-05-10

**Authors:** Zhiwei Sui, Xinhua Dai, Qingbin Lu, Yulan Zhang, Min Huang, Shufen Li, Tao Peng, Jie Xie, Yongzhuo Zhang, Chunchen Wu, Jianbo Xia, Lianhua Dong, Jiayi Yang, Wenfeng Huang, Siyuan Liu, Ziquan Wang, Ke Li, Qingfang Yang, Xi Zhou, Ying Wu, Wei Liu, Xiang Fang, Ke Peng

**Affiliations:** 1 Center for Advanced Measurement Science, National Institute of Metrology, Beijing, China; 2 Department of Laboratorial Science and Technology, School of Public Health, Peking University, Beijing, China; 3 State Key Laboratory of Virology, Wuhan Institute of Virology, Center for Biosafety Mega-Science, Chinese Academy of Sciences, Wuhan, Hubei China; 4 Joint Laboratory of Infectious Diseases and Health, Wuhan Institute of Virology and Wuhan Jinyintan Hospital, Wuhan, China; 5 Department of Laboratory Medicine, Huazhong University of Science and Technology, Tongji Hospital, Tongji Medical College, HUST, Wuhan, Hubei China; 6 Department of Laboratory Medicine, Maternal and Child Health Hospital of Hubei Province, Tongji Medical College, Huazhong University of Science and Technology, Wuhan, China; 7 Shenzhen Mindray Bio-Medical Electronics Co., Ltd., Shenzhen, China; 8 University of Chinese Academy of Sciences, Beijing, China; 9 State Key Laboratory of Virology / Institute of Medical Virology and School of Basic Medical Sciences, Wuhan University, Wuhan, China; 10 Key Laboratory of Pathogen and Biosecurity, Beijing Institute of Microbiology and Epidemiology, Beijing, China

**Keywords:** Immunology, Microbiology

## Abstract

Over 40% of the coronavirus disease 2019 (COVID-19) COVID-19 patients were asymptomatically infected with severe acute respiratory syndrome coronavirus 2 (SARS-CoV-2) and the immune responses of these asymptomatic individuals is a critical factor for developing the strategy to contain the COVID-19 pandemic. Here, we determined the viral dynamics and antibody responses among 143 asymptomatic individuals identified in a massive screening of more than 5 million people in eight districts of Wuhan in May 2020. Asymptomatic individuals were admitted to the government-designated centralized sites in accordance with policy. The incidence rate of asymptomatic infection is ~2.92/100,000. These individuals had low viral copy numbers (peaked at 315 copies/mL) and short-lived antibody responses with the estimated diminish time of 69 days. The antibody responses in individuals with persistent SARS-CoV-2 infection is much longer with the estimated diminish time of 257 days. These results imply that the immune responses in the asymptomatic individuals are not potent enough for preventing SARS-CoV-2 re-infection, which has recently been reported in recovered COVID-19 patients. This casts doubt on the efficacy of forming “herd-immunity” through natural SARS-CoV-2 infection and urges for the development of safe and effective vaccines.

## Introduction

As of December 23, 2020, the coronavirus disease 2019 (COVID-19) pandemic, caused by SARS-CoV-2 infection, has affected more than 78 million people leading to over 1.7 million death cases around the world. Besides COVID-19 patients with mild or severe respiratory illness, over 40% of individuals undergo asymptomatic infection without showing any symptoms.^1,2^ These asymptomatic individuals can efficiently transmit viral infection accounting for more than 30% of virus infection.^3,4^ These unnoticed and un-tractable transmission events have caused difficulty in controlling the COVID-19 pandemic.^5^

Lockdown on social activities during the COVID-19 pandemic has severely affected the world economy and people’s mental and physical health. In the absence of an effective vaccine or anti-viral drug against SARS-CoV-2, herd immunity, a strategy of allowing the coronavirus to spread until most people of the population become immune protected, has been proposed.^6^ Analysis suggests this would lead to the development of infection-acquired population immunity in the low-risk population, which will eventually protect the vulnerable people through adopting “focused protection”.^7^ While being endorsed by a number of scientists, these proposals have received criticism of being “scientifically and ethically problematic” and may lead to large numbers of unnecessary deaths. It is estimated that to reach the herd immunity, about 60–75% of the population need to be infected for attaining the population immunity. This would lead to millions of extra COVID-19 deaths and long-term complications that are difficult to be estimated.

Another critical factor for consideration when discussing application of “herd immunity” is the duration of protective immune responses in people infected by SARS-CoV-2. It has been reported that the protective immune responses among recovered COVID-19 patients last around 7 months.^8^ It is known that infection by seasonal coronaviruses is often associated with short-lived immune responses which can lead to multiple re-infection among the population.^9^ Similarly re-infection has been reported in recovered COVID-19 patients, some of whom experienced worse symptoms.^10–13^ This has cast much attention to the asymptomatic infected individuals whose anti-SARS-CoV-2 immune responses would be a critical determinant for the population immunity considering the potentially large number of these individuals.

Here, we measured the viral dynamics using patient saliva samples^14^ and antibody responses using serum samples in asymptomatically infected individuals. It is found that the viral copy number is low among these individuals and the antibody responses are short-lived lasting about 69 days. In comparison, patients with persistent SARS-CoV-2 infection maintained antibody responses lasting around 257 days. Results from this study indicate that antibody responses among asymptomatic individuals may not be potent and persistent enough to prevent these people from SARS-CoV-2 re-infection. This argues against application of the strategy of “herd immunity” and urges development of effective vaccines and anti-viral drugs.

## Results

### The demographic characteristics of two cohorts of SARS-CoV-2 infection

#### Cohort 1 of asymptomatic SARS-CoV-2 infection

**Fig. 1:**
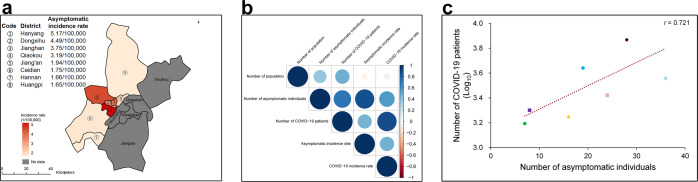
The detection of asymptomatic individuals with SARS-CoV-2 infection and correlation with case number. **a** The incidence rates of asymptomatic patients in eight districts of Wuhan city; **b** Correlation matrix of asymptomatic individuals with total population and total COVID-19 patients. The number of COVID-19 patients was log10-transformed. The size and the color of each dot in the triangular matrix show the strength of Pearson correlation (positive or negative) between the variables. Positive strong correlations stand out in dark blue, lighter colors indicate weaker correlations; **c** The correlation of number of asymptomatic individuals with the number of COVID-19 patients (log10-transformed) in the eight districts of Wuhan city and the linear fitting was performed. The different color and dots represent for the eight different districts of Wuhan city (green dot for Hannan District, purple dot for Caidian District, yellow dot for Huangpi District, light blue dot for Jiangan District, dark red dot for Dongxihu District, black dot for Jianghan District, light red dot for Qiaokou District and greyish blue dot for Hanyang District)

In the screening of SARS-CoV-2 positive individuals in eight districts of Wuhan city, a total 5,685,100 individuals were tested and 166 asymptomatic SARS-CoV-2 positive individuals were identified through RT-PCR tests, yielding an incidence rate of 2.92/100,000 (166/5,685,100). The highest incidence rate was observed in the Hanyang District (5.17/100,000), followed by 4.49/100,000 in the Dongxihu District and 3.75/100,000 in the Jianghan District (Fig. 1a). On the district level, the number of asymptomatic individuals was significantly correlated with the case number of reported COVID-19 patients (coefficient efficiency *r* = 0.721, *P* = 0.044), more closely than that with the incidence rate of COVID-19 patients (*r* = 0.568, *P* = 0.143) (Fig. 1b, c). A total of 143 asymptomatic individuals with SARS-COV-2 were recruited in the study. Their mean (±SD) age was 50 (±16) years old and 63 (44.1%) were male, which were similar to the general COVID-19 patients in China (the mean age were 51 years; 51.4% of the COVID-19 patients were male, *P* = 0.078) (Table 1).^15^ Among these asymptomatic carriers, 34 (24.5%) patients had underlying diseases, including 19 (13.3%) with hypertension and 10 (7.0%) with diabetes, which are both comparable with the proportion of all the COVID-19 patients (12.8% and 5.3% respectively, both *P* > 0.05). This indicates that the comorbidity is not an impacting factor for being asymptomatic carrier of SARS-CoV-2.

**Table 1:** Basic information of the asymptomatic patients with SARS-COV-2 infection in the study

Characteristics	Asymptomatic (*n* = 143)	Persistent infection (*n* = 20)	*P*
Age, years, mean ± SD	50 ± 16	58 ± 12	0.033
≤45	49 (34.3)	3 (15.0)	0.100
45–60	53 (37.1)	7 (35.0)	
>60	41 (28.7)	10 (50.0)	
Sex, *n* (%)			0.009
Male	63 (44.1)	15 (75.0)	
Female	80 (55.9)	5 (25.0)	
Underlying diseases, *n* (%)	36 (25.2)	11 (55.0)	0.006
Hypertension	19 (13.3)	7 (35.0)	0.013
Diabetes	10 (7.0)	3 (15.0)	0.202

Underlying diseases include chronic viral hepatitis, chronic obstructive pulmonary diseases, chronic cardiovascular diseases, and chronic heart diseases

*SD* standard deviation, *SARS-COV-2* severe acute respiratory syndrome coronavirus 2

#### Cohort 2 persistent SARS-CoV-2 infection

A group of 20 patients who were identified to be SARS-CoV-2 positive for more than 30 days were recruited as cohort 2 for comparison. Among these patients the mean (±SD) age was 58 (±12) years old and 15 (75.0%) were male. Statistical analysis revealed that persistent SARS-CoV-2 infection was more frequently observed in patients with older age (*P* = 0.017) and in male patients (*P* = 0.026) (Table 1).

### Dynamic profiles of anti-SARS-COV-2 antibody in the cohort of asymptomatic SARS-CoV-2 infection

**Fig. 2:**
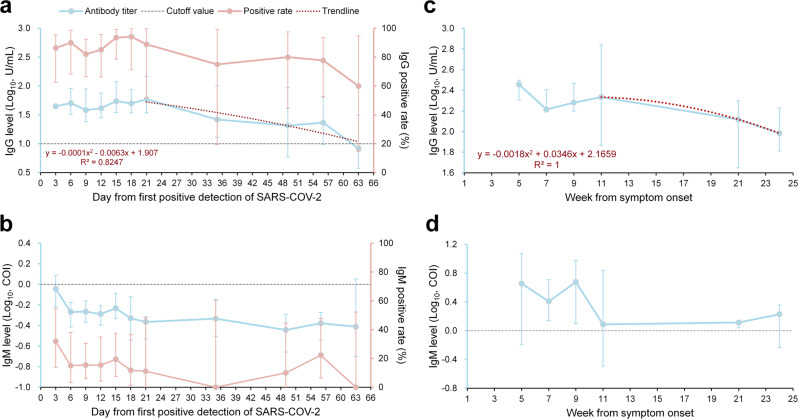
Dynamic profiles of IgG and IgM antibody titers and positive rates in the asymptomatic individuals and persistent COVID-19 patients with SARS-CoV-2 infection. **a** Dynamic profiles of IgG antibody titers (blue line, mean and standard deviation) and positive rates (pink line, rate and 95% confidence interval) of the asymptomatic individuals with SARS-CoV-2 infection. The quadratic fitting curves were performed for IgG antibody titers in the decaying stage; **b** Dynamic profiles of IgM antibody titers (blue line, mean and standard deviation) and positive rates (pink line, rate and 95% confidence interval) of the asymptomatic individuals with SARS-CoV-2 infection; **c** Dynamic profiles of IgG antibody titers (blue line, median and interquartile range) of the patients with persistent SARS-CoV-2 infection. The quadratic fitting curves were performed for IgG antibody titers in the decaying stage; **d** Dynamic profiles of IgM antibody titers (blue line, median and interquartile range) of the patients with persistent SARS-CoV-2 infection

The anti-SARS-CoV-2 IgG antibody level was plotted for the Cohort 1 of asymptomatic SARS-CoV-2 infection every 3 days (Fig. 2a). The positive rate started at 86.4% (95% CI 62.6–95.3%) for the first sampling point, with the peaking level reached 94.1% (95% CI 71.3–99.9%) at Day 18 after the first positive detection. The IgG level started at 44.4 (95% CI 28.7–69.6) U/mL for the first sampling point, with the peaking level reached 58.8 (95% CI 29.0–119.3) U/mL at Day 21 after first positive detection. Thereafter both levels decreased slowly until Day 63 (positive rate 60%, 95% CI 14.7–94.7%; IgG level 8.3, 95% CI 0.7–99.3), which was the last sampling date. The quadratic fitting curve was plotted for the IgG antibody titer at the decay stage after Day 21 (*R*^2^ = 0.825), based on which the half-life of IgG was estimated as ≈25 days and the diminishing time to vanish was estimated as 69 days after the first positive detection of SARS-CoV-2. The anti-SARS-CoV-2 IgM response profile for the asymptomatic patients with SARS-CoV-2 infection was also plotted every 3 days (Fig. 2b). In a different manner from IgG, the IgM had the highest positive rate at Day 3 (31.8%, 95% CI 13.9–54.9%) and then fluctuated till all negative to the end of observation. The GMRT of IgM antibody titer was below the cutoff index (1 COI) during the studied period.

### Dynamic profiles of anti-SARS-CoV-2 antibody in the cohort of persistent SARS-CoV-2 infection

The anti-SARS-CoV-2 IgG antibody was plotted for the persistent SARS-CoV-2 infection patients every 2 weeks (Fig. 2c). Based on the IgG level quantification, the IgG level was the highest at Week 5 after the first disease onset (also the first sampling of the current episode of SARS-CoV-2 detection) (IgG level 285.8 U/mL, 95% CI 202.3–314.5), maintained at comparable levels until Week 11, followed by persistent decreasing to the end of observation. The quadratic fitting curve was plotted on the IgG antibody titers during the decay stage after Week 11 (*R*^2^ = 1.000), based on which, the half-life of IgG was estimated at ≈36 days and the diminish time was estimated at about 257 days (≈9 months) post-symptom onset. Similarly with IgG, the IgM titer maintained at a comparable level till Week 9 (IgM level 4.7 COI, 95% CI 1.3–9.5), followed by a decrease to low level at Week 11 and a persistent plain level to the end of observation (Fig. 2d). Comparison between two cohorts revealed significantly higher antibody level in the Cohort 2 than in Cohort 1 for both IgG (OR = 2.269, 95% CI 1.808–2.848, *P* < 0.001) and IgM (OR = 2.602, 95% CI 2.239–3.024, *P* < 0.001) after adjusted the variables of age, sex and underlying diseases by GEE. These differences potentially represented different viral replication status.

### The impacting factor of antibody level in the two cohorts

For the asymptomatic infection, the GEE based on multiple measurements of IgG level and positive rate during the observation, revealed that higher IgG antibody titer was observed in the patients aged 45–60 years old (OR = 1.255, 95% CI 1.015–1.555) and the IgG titer was the highest in individuals of >60 years old (OR = 1.422, 95% CI 1.113–1.817). GEE based on IgM evaluation disclosed no effect from age, sex, or underlying diseases on the positive rate. However, female patients had a higher IgM antibody titer level than the male patients (OR = 1.141, 95% CI 1.022–1.272). Both IgG and IgM levels decreased with the increasing days from the first detection of SARS-CoV-2 (OR = 0.995, 95% CI 0.993–0.997; OR = 0.996, 95% CI 0.994–0.998) (Table 2). For the patients with persistent SARS-CoV-2 infection, the patients of >60 years old had a higher level of IgG antibody titer (OR = 1.404, 95% CI 1.090–1.807), similar with the asymptomatic individuals, while the patients with underlying diseases (OR = 0.651, 95% CI 0.492–0.862) and more days from symptom onset to antibody detection (OR = 0.997, 95% CI 0.994–0.999) had lower levels of IgG antibody titer (Table 3).

**Table 2:** The related factors associated with IgG and IgM antibody levels and positive rates of the asymptomatic individuals with SARS-CoV-2 infection by generalized estimating equation

Variable	Positive rate				Antibody titers			
	IgG OR (95% CI)	*P*	IgM OR (95% CI)	*P*	IgG OR (95% CI)	*P*	IgM OR (95% CI)	*P*
Age, years								
>60	1.110 (0.948–1.299)	0.194	0.97 (0.817–1.152)	0.728	1.422 (1.113–1.817)	0.005	1.098 (0.946–1.276)	0.22
45–60	1.104 (0.963–1.265)	0.155	1.009 (0.869–1.172)	0.906	1.255 (1.015–1.553)	0.036	1.054 (0.925–1.201)	0.431
≤45	Reference		Reference		Reference		Reference	
Sex								
Female	1.120 (0.999–1.255)	0.052	1.041 (0.918–1.180)	0.530	1.142 (0.955–1.365)	0.145	1.141 (1.022–1.272)	0.018
Male	Reference		Reference		Reference		Reference	
Underlying diseases								
Yes	1.141 (0.988–1.318)	0.073	0.953 (0.815–1.114)	0.546	1.245 (0.993–1.561)	0.057	0.957 (0.834–1.097)	0.525
No	Reference		Reference		Reference		Reference	
Days from the first detection of SARS-COV-2	0.999 (0.997–1.000)	0.155	1 (0.997–1.002)	0.680	0.995 (0.993–0.997)	<0.001	0.996 (0.994–0.998)	<0.001

*OR* odds ratio, *CI* confidence interval, *SARS-CoV-2* severe acute respiratory syndrome coronavirus 2

**Table 3:** Multivariate analysis on the factors related to the IgG/IgM antibody levels of the patients with persistent SARS-CoV-2 infection

Characteristics	IgG		IgM	
	OR (95% CI)	*P*	OR (95% CI)	*P*
Age, years				
>60	1.404 (1.090–1.807)	0.009	0.878 (0.517–1.491)	0.631
≤60	Reference		Reference	
Sex				
Female	1.272 (0.968–1.672)	0.084	0.671 (0.374–1.206)	0.182
Male	Reference		Reference	
Underling diseases				
Yes	0.651 (0.492–0.862)	0.003	1.093 (0.634–1.884)	0.748
No	Reference		Reference	
Duration for SARS-CoV-2 positive, days				
>60	1.037 (0.796–1.352)	0.789	0.885 (0.531–1.476)	0.641
≤60	Reference		Reference	
Days from symptom onset to antibody detection	0.997 (0.994–0.999)	0.019	0.996 (0.995–0.997)	<0.001

The multivariate analysis was performed for IgG/IgM antibody levels by generalized estimating equation

*OR* odd ratio, *CI* confidence interval, *COVID-19* coronavirus disease 2019, *SARS-CoV-2* severe acute respiratory syndrome coronavirus 2

### Dynamic profile of viral loads in the asymptomatic patients with SARS-CoV-2 infection

**Fig. 3:**
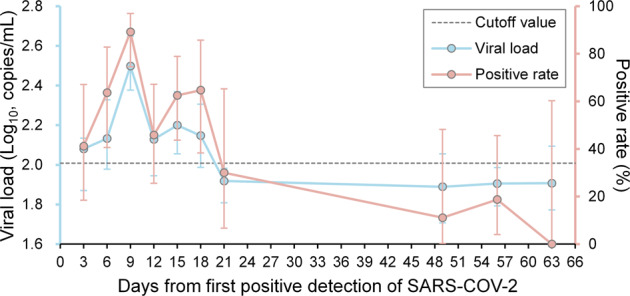
Dynamic profile of viral loads and positive rates in asymptomatic individuals with SARS-CoV-2 infection. The blue line represents dynamic profile of viral loads (mean and standard deviation) based on N gene of SARS-CoV-2 in the saliva samples. The pink line represents dynamic profile of positive rates (rate and 95% confidence interval)

**Fig. 4:**
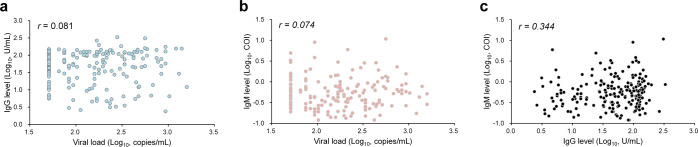
The scatter plots among the IgG and IgM titers and viral loads based on N gene in the asymptomatic individuals with SARS-CoV-2 infection. **a** The correlation between IgG level and viral loads; **b** The correlation between IgM level and viral loads; **c** The correlation between IgG level and IgM level. The correlation coefficient was calculated by Spearman rank correlation analysis

The dynamic profiles of viral loads and positive rate of SARS-CoV-2 in the samples of saliva from the Cohort 1 were illustrated in Fig. 3. The viral loads of SARS-CoV-2 based on N gene in saliva peaked at Day 9 of the first detection (315.1 copies/mL, 95% CI 238.1–417.1), followed by gradual decrease and from Day 21 detection was below the cut-off value (102 copies/mL) in Fig. 3. The positive rate of SARS-CoV-2 detection showed the same trend with the viral loads. The viral loads of SARS-CoV-2 were compared regarding age, sex, underlying diseases and interval days from first test, which demonstrated that viral loads decreased with the days from first detection of SARS-CoV-2 (OR = 0.992, 95% CI 0.989–0.996; Supplementary Table 1). No significant associations were observed between other variables and the viral loads. The Spearman correlation analysis was performed among the viral loads based on N gene from saliva and IgG/IgM levels (Fig. 4). The correlations were low as all the correlation coefficients were lower than 0.500 among any two of viral loads and IgG/IgM levels, potentially due to the very low viral load that might not stimulate strong antibody responses.

## Discussion

It is known that the protective immunity for seasonal coronaviruses is short-lived and the anti-viral immune responses may not last long enough for protecting the host from re-infection.^9^ Here we report that individuals with asymptomatic SARS-CoV-2 infection have short-lived antibody responses lasting for only around 69 days. This duration is much shorter than that of the recovered COVID-19 patients or persistent COVID-19 patients whose IgG response lasts for around 211 days^8^ or 257 days, respectively. The short-lived immune responses in asymptomatic patients will likely raise the risk for these individuals being susceptible for SARS-CoV-2 re-infection, which has been reported in recovered COVID-19 patients.^10,12,13^ On the other hand, it might be difficult to track re-infection of asymptomatic patients as these individuals do not develop symptoms and would not be identified during the first infection. Since over 40% of people may experience asymptomatic infection with SARS-CoV-2,^1^ re-infection of asymptomatic individuals would cast concern to the rationality for the “herd immunity” strategy. Recently, more severe symptoms including death have been reported in the re-infection cases.^10,12^ This indicates that safe and effective vaccine program is vital for containing the COVID-19 pandemic.

Another factor to consider is the cellular immune responses among these asymptomatically infected individuals. However, due to the absence of blood cells in the available serum samples, it was not feasible to monitor the effector/memory T cells and cellular immunity in the asymptomatic individuals in this study. It was recently reported that the breadth and magnitude of T cell responses were significantly higher in severe as compared with mild cases, while higher proportions of SARS-CoV-2-specific CD8^+^ T cells were observed in mild cases.^16^ However, the comparison of T cell responses between asymptomatic individuals and symptomatic COVID-19 patients has not been reported by far. It would be more informative for future studies to monitor the cellular immunity in the asymptomatically infected individuals with convalescent COVID-19 patients or symptomatic patients with on-going infection.

The duration of anti-SARS-CoV-2 immune responses among COVID-19 patients appear to be correlated with the duration of virus infection. It is found in this study that patients with persistent SARS-CoV-2 infection have the longest duration of immune responses of 257 days which is longer than the recovered COVID-19 patients of about 211 days^8^ and much longer than the asymptomatic infection of 69 days. This suggests that a vaccination program with multiple stimulations might be more effective for inducing long-lasting anti-viral immune responses. Different from SARS-CoV-2, SARS-CoV infection may lead to long-lasting humoral immunity for 3 years in up to 50% of the patients.^17^ To further understand the longevity of anti-SARS-CoV-2 immune responses, it might be important to monitor the duration and neutralization activity of antibody responses among recovered COVID-19 patients and vaccinated population over longer periods of several years.

Notably, the massive community screening and epidemiological analysis showed that people who have close contact with these asymptomatic individuals were not SARS-CoV-2 positive in the RT-PCR testing. Also attempts to isolate virus from these asymptomatic individuals were unsuccessful. This indicates that despite being positive with SARS-CoV-2, these asymptomatic individuals may not be able to transmit the virus. Absolute quantitative analysis with reverse transcription digital polymerase chain reaction dPCR (RT-dPCR) analysis revealed that the viral copy numbers of these asymptomatic individuals are low with the peak level of around 315 copies/mL. The low SARS-CoV-2 copy number in these individuals may not be sufficient for virus transmission. Application with this quantification analysis might help to form the decision on how and for how long these asymptomatic individuals should be quarantined.

To our knowledge, this study presents by far the largest analysis of viral dynamics and antibody responses of asymptomatic individuals covering 143 patients. It would be interesting to compare whether the viral dynamics and antibody responses in asymptomatic individuals in other countries follow a similar trend. In any case, identification of asymptomatically infected individuals through community screening and social distancing would still be important measures for controlling the current COVID-19 pandemic.

## Materials and methods

### Study design and participants

All study participants were enrolled and sampled in accordance to the Medical Ethics Committee of Wuhan Infectious Disease Hospital (KY-2020-75.01) and was conducted from May 2020 to May 2021. Demographic characteristics, clinical data and samples were only collected after the study participant had acknowledged that they had understood the study protocol and signed the informed consent. All participant information and samples were collected in association with study identifiers.

Cohort 1 included 143 asymptomatic individuals recruited from 166 asymptomatic SARS-CoV-2 positive individuals who were identified through mass screening in eight districts of Wuhan city in May 2020, and were asked to stay in medical isolation observation for a further 14 days at government-designated hotels, in a single room for each patient. Asymptomatic individuals were defined as their SARS-CoV-2 RNA is detectable but symptoms never develop during the study period from the date of diagnosis.

Cohort 2 included 20 patients with persistent SARS-CoV-2 infection who were enrolled from Wuhan Jinyintan Hospital. A retrospective cohort study was conducted at Wuhan Jinyintan Hospital in Wuhan city from February 1st to April 24th, 2020. All these patients with confirmed infection were isolated in solitary. During the observation, symptom monitoring was performed via daily collection of body temperature and any clinical symptoms that were related to SARS-CoV-2 infection. As guided by the China Centers for Disease Control and Prevention, a negative conversion of RT-PCR assay for SARS-CoV-2 was defined as negative results from respiratory tract samples, the end of isolation was taken after consecutive negative results 24 h apart after 1–2 weeks from isolation. Then after isolation, the follow-up investigation was performed.

### Sample collection

Blood samples and saliva samples were collected from in all patients of two cohorts at designated time points by trained healthcare workers. The serum samples were separated after centrifugation at 3000 rpm and then inactivated at 56 °C for 30 min. For the collection of saliva samples, patients were asked to place a piece of cotton from salivette (SARSTEDT, 51.1534) into their mouth without chewing before washing their hands and to spit the cotton back to the salivette after 2 min.

### Serum anti-SARS-CoV-2 antibodies assay

Total SARS-CoV-2 IgM or IgG in the serum was measured by chemiluminescence using commercially available kits (Shenzhen Mindray Bio-medical Electronics Co. Ltd) in all patients of two cohorts at different time points. The magnetic beads of this kit are coated with nucleoprotein (N protein) and receptor-binding domain of the spike protein (S protein) of SARS-CoV-2. Briefly, IgG and IgM detections consist of two steps. In the first step, antigen-coated paramagnetic microparticles captured antibody in the sample specific to the antigens. After a wash step to remove unbound substances, anti-human IgG/IgM antibody conjugated alkaline phosphatase was added to bind to antibodies captured by paramagnetic microparticles. After the second wash step to remove unbound substances, 3-(2′-spiroadamantyl)-4-methoxy-4-(3″- phosphoryloxy)-phenyl-1,2-dioxetane (AMPPD) was added and catalyzed by alkaline phosphatase to emit light at 540 nm. The resulting chemiluminescent reaction was measured as relative light unites (RLUs) by a photomultiplier in the chemiluminescence immunoassays analyzer (CL-6000i) (Shenzhen Mindray Bio-medical Electronics Co. Ltd). IgG antibodies were calculated as U/ml and the Cutoff value is 10 U/ml. IgM antibodies were presented as the measured RLU divided by the cutoff (cutoff index, COI): COI ≥ 1 was defined as positive and COI < 1 as negative. The IgG kits and IgM kits were CE marked.

### RNA extraction and SARS-CoV-2 detection via RT-dPCR

In brief, 300 μL of liquid of each specimen was applied for RNA extraction using nucleic acid extraction kit (Liferiver, Z-ME-0044) following the manufacturer’s instructions with an automatic nucleic acid extractor (Liferiver, EX3600/2400). After extraction, the total nucleic acid was recovered using 70 μL of elution buffer. For SARS-CoV-2 RNA detection, 5 μL of RNA template was tested using one step RT-dPCR assay targeting nucleocapsid protein (N) gene, as we described previously.^18^ The cycled plate was then transferred to the QX200 Droplet Reader (Bio-Rad) and analyzed using the QuantaSoft droplet reader software (V1.7.4, Bio-Rad). Reactions containing more than 10,000 droplets were treated as effective and involved in data analysis. The determination of a positive result should meet the following criteria: quantification of N gene target is ≥2.2 copies/reaction.

### Statistical analyses

Categorical variables were expressed as frequencies and proportions, and continuous variables were expressed as medians and interquartile ranges or means and standard deviations (SDs). Proportions for categorical variables were compared using *χ*^2^ or Fisher exact test. Continuous variables were analyzed using the Mann–Whitney *U* test method. To estimate marginal effects and linear time interaction by group, generalized estimating equations were used to compare appropriacy of dynamics of viral loads and IgG/IgM levels between groups. Pearson or Spearman correlation analysis was performed to explore the correlations among different variables. All statistical analyses were performed using Stata 14.0 (Stata Corp LP, College Station, TX, USA). Statistical significance was set as *P* < 0.05.

## Supplementary information

Supplementary Materials

## Data Availability

The data sets used for the current study are available from the corresponding author upon reasonable request.
