# Supplementary material for: Viral dynamics and antibody responses in people with asymptomatic SARS-CoV-2 infection
Source: Signal Transduct Target Ther. 2021 May 10;6:181. doi: 10.1038/s41392-021-00596-2 (PMC8107204; doi:10.1038/s41392-021-00596-2)
Supplement: Supplementary file 1 — Supplementary Materials [file 41392_2021_596_MOESM1_ESM.docx]

Supplementary Materials for

Viral dynamics and antibody responses in people with asymptomatic SARS-CoV-2 infection

Zhiwei Sui, Xinhua Dai, Qin-Bin Lu, Ms Ying Liu, Yulan Zhang, Shufen Li, Zhenjin Ke, Ke Hong, Zhenghui Huang, Tao Peng, Jie Xie, Yongzhuo Zhang, Chunchen Wu, Jianbo Xia , Lianhua Dong, Jiayi Yang, Wenfeng Huang, Siyuan Liu, Ziquan Wang, Ke Li, Qingfang Yang, Ying Wu, Xi Zhou, Dingyu Zhang, Chaolin Huang, Wei Liu, Xiang Fang, Ke Peng.

Correspondence to: pengke@wh.iov.cn

**This PDF file includes:**

Tables. S1

Tables. S1

| Variable | Viral load | |  | Positive rate | |
| --- | --- | --- | --- | --- | --- |
|  | OR (95% CI) | P |  | OR (95% CI) | P |
| Age, years |  |  |  |  |  |
| >60 | 0.928 (0.792-1.088) | 0.358 |  | 0.986 (0.813-1.195) | 0.883 |
| 45-60 | 1.051 (0.914-1.209) | 0.484 |  | 1.085 (0.915-1.286) | 0.346 |
| ≤45 | Reference |  |  | Reference |  |
| Sex |  |  |  |  |  |
| Female | 1.022 (0.909-1.148) | 0.720 |  | 0.948 (0.822-1.092) | 0.459 |
| Male | Reference |  |  | Reference |  |
| Underlying diseases |  |  |  |  |  |
| Yes | 1.006 (0.876-1.156) | 0.928 |  | 1.012 (0.855-1.198) | 0.888 |
| No | Reference |  |  | Reference |  |
| Days from first detection of SARS-COV-2 | 0.992 (0.989-0.996) | <0.001 |  | 0.989 (0.985-0.993) | 0.012 |

**Supplemental Table 1. The related factors associated with viral loads and positive rates in saliva of the asymptomatic individuals with SARS-CoV-2 infection by generalized estimating equation.**

OR, odds ratio. CI, confidence interval. SARS-CoV-2, severe acute respiratory syndrome coronavirus 2.
